# Goldenhar syndrome with blepharophimosis and limb deformities: a case report

**DOI:** 10.1186/s12886-018-0872-5

**Published:** 2018-08-22

**Authors:** Xia Ding, Xi Wang, Yuan Cao, Jiaying Zhang, Ming Lin, Xianqun Fan, Jin Li

**Affiliations:** 1 Department of Ophthalmology, Ninth People’s Hospital, Shanghai JiaoTong University School of Medicine, No.639 ZhizaojuRoad, Shanghai, 200025 People’s Republic of China; 2 Shanghai Key Laboratory of Orbital Diseases and Ocular Oncology, Shanghai, China

**Keywords:** Goldenhar syndrome, Blepharoptosis, Limb deformities

## Abstract

**Background:**

Goldenhar syndrome has variable presentations and can affect multiple regions of the body. Diagnoses are based on clinical manifestations. The association of Goldenhar syndrome with blepharophimosis and limb deformities has not previously been reported. Here, we describe a patient who was diagnosed with Goldenhar syndrome in association with blepharophimosis, ocular hypertelorism, hearing loss and limb deformities.

**Case presentation:**

A 10-year-old male was first referred to our ophthalmology clinic on 2009–2-11 for ocular hypertelorism and microphthalmia when he had chin-up position. In the first ophthalmic examination, his palpebral fissure length was 19 mm on the right and 20 mm on the left, both palpebral fissure height was 4 mm, the inner intercanthal distance was 63 mm, both upper margin reflex distances were − 1 mm, the myodynamia of the levator palpebrae muscle was 2 mm on the right and 3 mm on the left, and his visual acuity was 20/40 on the right and 20/32 on the left. A physical examination revealed the patient had developed limb deformities in his hands, wrists, elbows and shoulders along with hearing loss. The patient was diagnosed with Goldenhar syndrome because his clinical presentations included ocular hypertelorism, hearing loss, and multiple acral joint deformities. He underwent a first operation in 2009 and a second in 2015. The second operation achieved a satisfactory result in which the horizontal fissure length was 28 mm on both sides, both palpebral fissure height was 10 mm, the inner intercanthal distance was 30 mm, and both of the upper margin reflex distances were 4 mm. He continued to wear hearing aids as usual. His hearing loss and joint deformities were slated for long-term follow-up at his parents’ request.

**Conclusion:**

The patient, diagnosed with Goldenhar syndrome in association with blepharophimosis, ocular hypertelorism, hearing loss and limb deformities, underwent two operations and achieved a satisfactory result. The patient was submitted to long-term follow-up observations and symptomatic treatments that vary with age and systemic associations, as needed. When treating patients with Goldenhar syndrome, ophthalmology specialists should cooperate with a multi-disciplinary team of clinicians and reach agreement regarding the appropriate systemic and comprehensive treatments.

## Background

Goldenhar syndrome (GS), also known as oculo-auriculo-vertebral dysplasia, was first described by Von Arlt in 1854 and was named after Dr. Maurice Goldenhar, who reported 3 cases in 1952. In craniofacial malformations that occur during the foetal period, the incidence of GS ranks second only to cheilopalatognathus. The prevalence of GS ranges from 1:4500 to 1:5600 live births and has a male-to-female ratio of 3:2 [1]. There is no agreement in the literature regarding the diagnostic criteria for GS. Currently, a diagnosis depends on clinical manifestations. In particular, GS presents with multiple malformations and various clinical manifestations [2], including ocular abnormalities; aural, facial, and spinal dysplasia and other systemic abnormalities [3].

## Case presentation

A 10-year-old boy was admitted to the Department of Ophthalmology on 2009–2-11 for ocular hypertelorism and microphthalmia when he had chin-up position. Over the past ten years, neither the inability of the patient to fully open his eyes nor his ocular hypertelorism had improved. He underwent a first operation in our department in 2009 and a second in 2015. The parents were healthy, and their marriage was non-consanguineous. The parents denied a family history of pertinent causes, such as genetic abnormalities or infections.

**Fig. 1:**
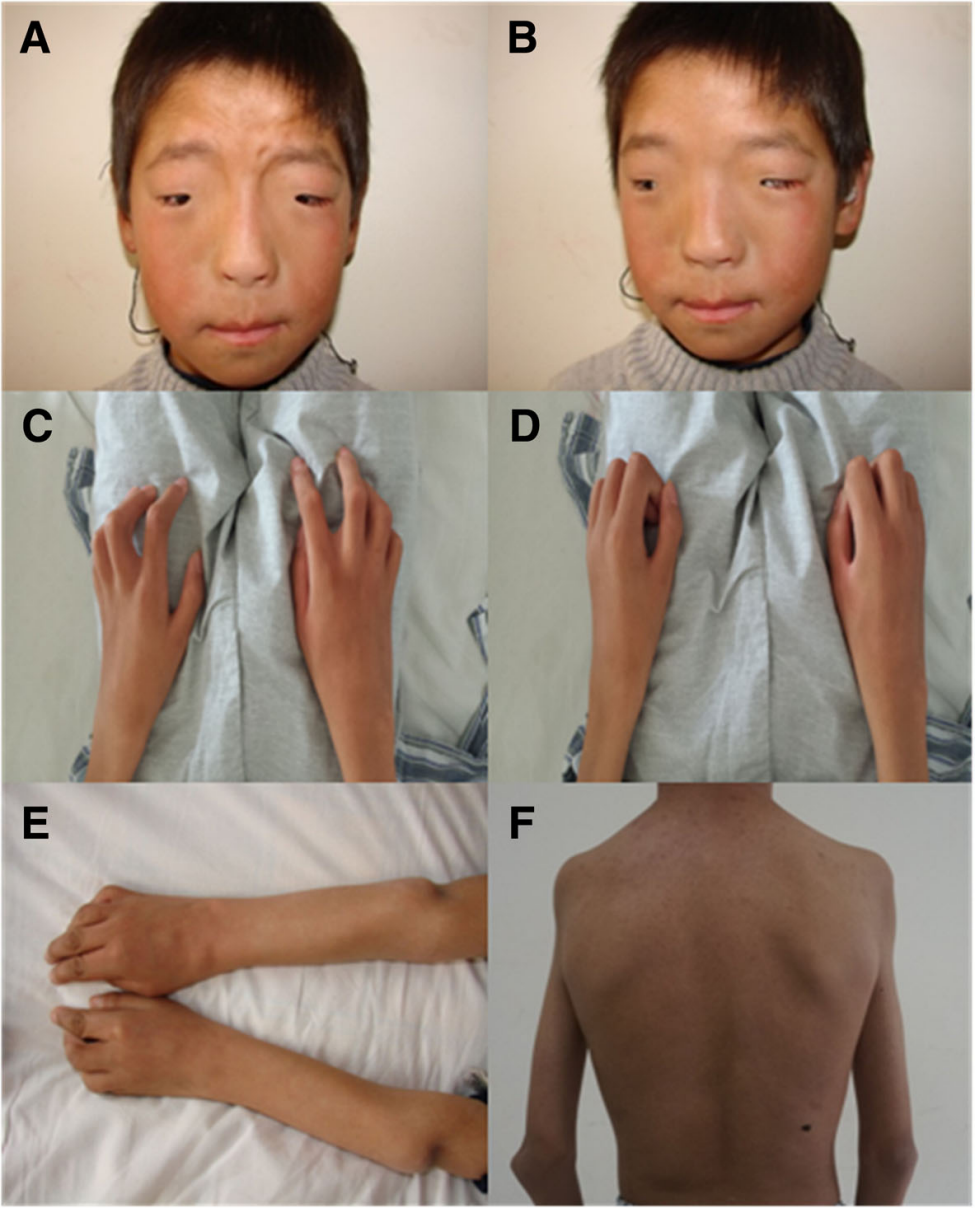
An examination performed before surgery showed the following: **a** and **b**: ocular hypertelorism and blepharophimosis. **c** and **d**: the bilateral finger joints were flexed and could not be extended, and the patient could not clench his fists. **e**: elbow malformations. **f**: shoulder malformations

**Fig. 2:**
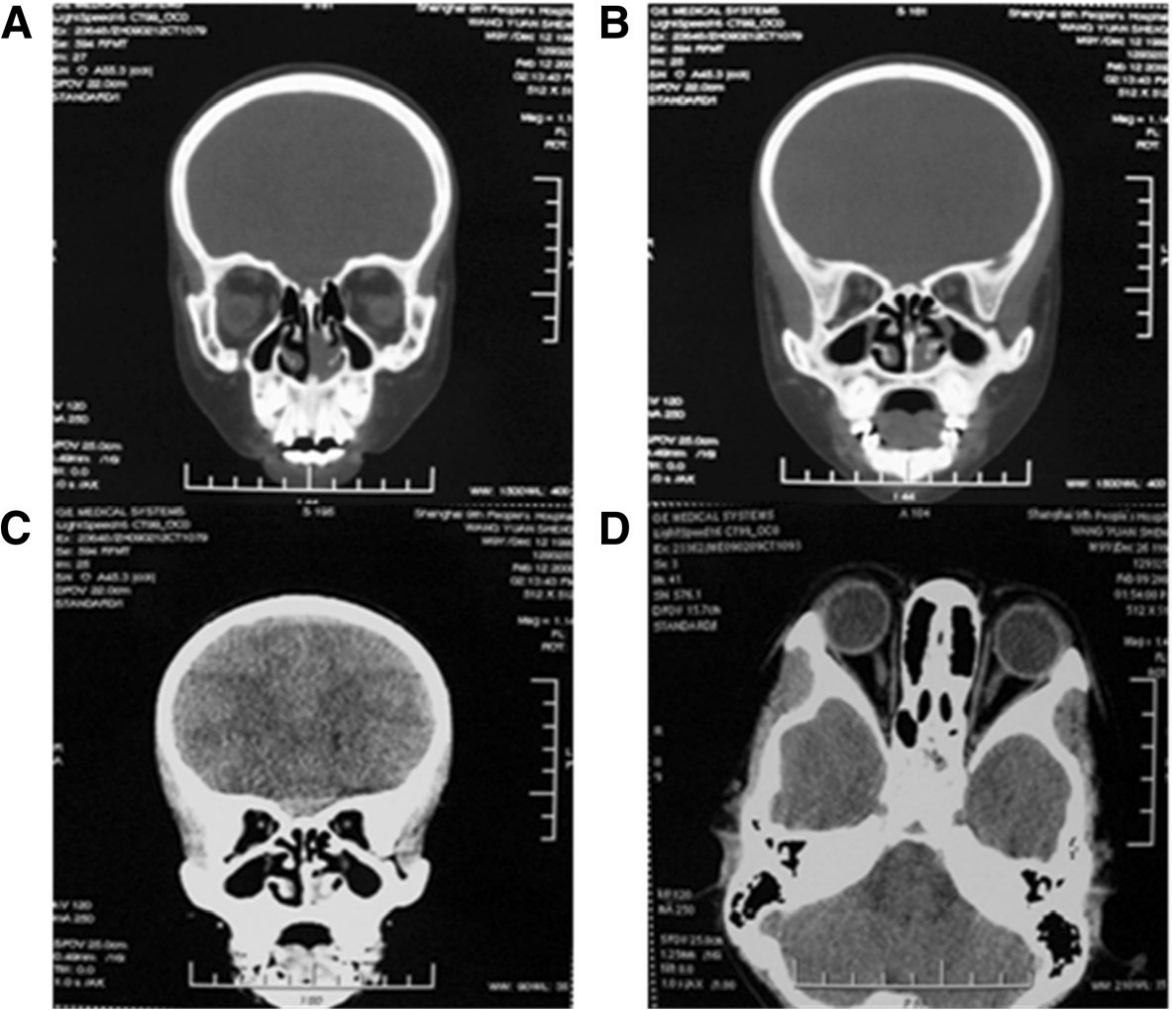
**a**-**d** An orbital CT performed in 2009 showed the following: symmetrical line sag in the bilateral anterior maxillary sinuses with normal bone structures in the orbit

In the original ophthalmic examination, the palpebral fissure length was 19 mm on the right side and 20 mm on the left side, both palpebral fissure height was 4 mm, the inner intercanthal distance was 63 mm, both upper margin reflex distances were − 1 mm, and the myodynamia of the levator palpebrae muscle was 2 mm on the right and 3 mm on the left. The patient had no conjunctival congestion, a transparent cornea, and a normal retina (Fig. 1a and b). The patient’s visual acuity was 20/40 in the right eye and 20/32 in the left eye. An orbital CT scan revealed symmetrical line sag in the bilateral anterior maxillary sinuses, increased density in the bilateral maxillary sinuses, no obvious abnormal orbital structures and no bone destructions (Fig. 2a-d).

The results of a physical examination revealed that the patient had developed bilateral limb deformities in his hands, wrists, elbows and shoulders. The bilateral interphalangeal finger joints were flexed and could not be extended, and the metacarpophalangeal joints and wrists exhibited a limited range of motion. The patient could not clench his fists or fully extend his elbows, which exhibited subluxation (Fig. 1c-1e). He could not fully extend his shoulders, and he exhibited square shoulders and weak adduction (Fig. 1f). He had worn hearing aids (prescribed by a local hospital to treat his sensorineural hearing loss) for 9 years. No intellectual disability was detected during the examination.

**Fig. 3:**
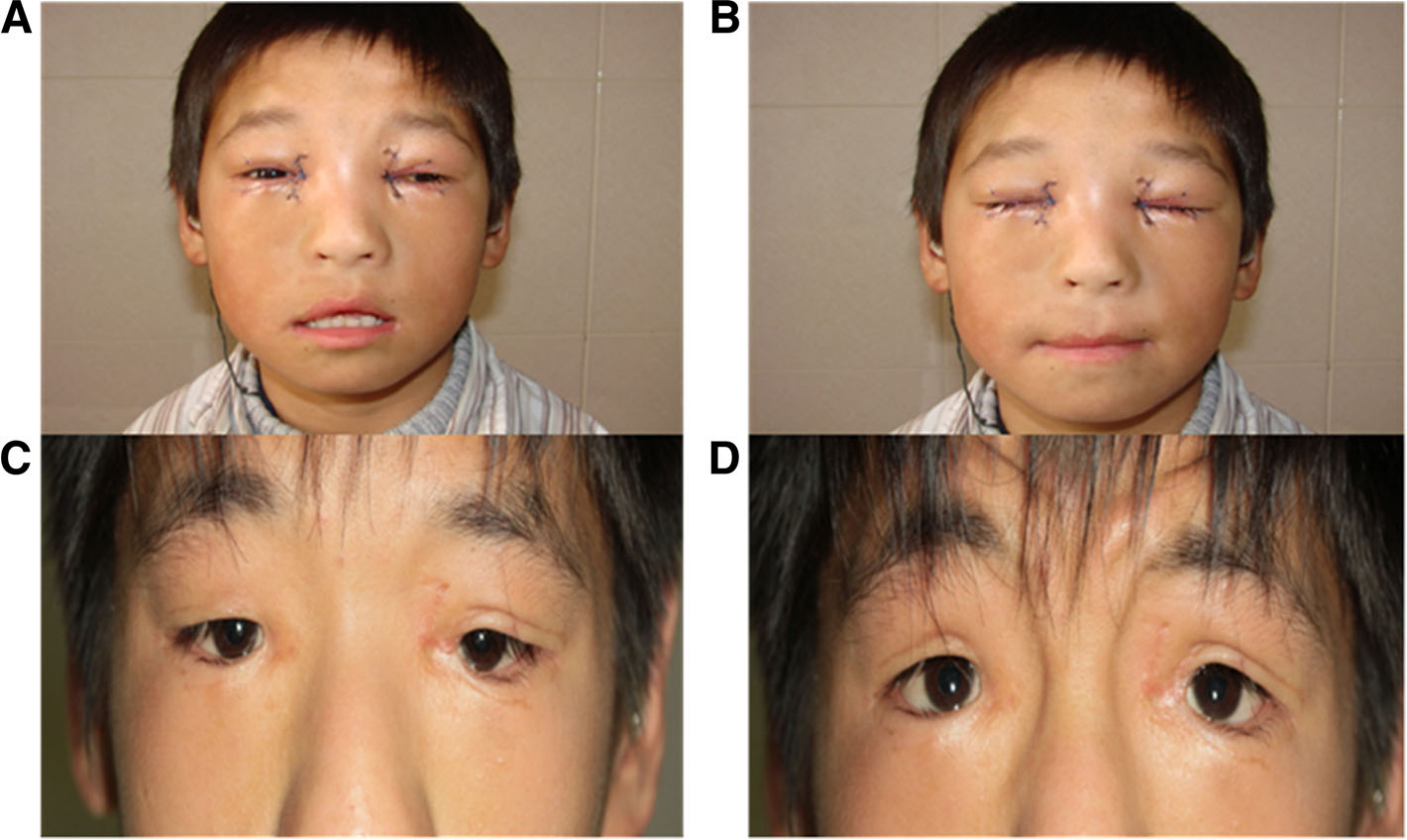
Postoperative examinations showed the following: **a** and **b**: A postoperative examination performed in 2009 showed that the palpebral fissure length was 28 mm on the right and 27 mm on the left, both palpebral fissure height was 8 mm, the inner intercanthal distance was 40 mm, and both of the upper margin reflex distances were 3 mm. **c** and **d**: A postoperative examination performed in 2015 showed that the palpebral fissure length was 21 mm on the right and 22 mm on the left, both palpebral fissure height was 6 mm, the inner intercanthal distance was 48 mm, and both of the upper margin reflex distances were 2 mm

The patient wanted to achieve symmetrical and natural-looking cosmetic results. The ophthalmologists performed inner canthus moulding combined with blepharoptosis correction in 2009. The surgical procedure included medial canthoplasty performed using the Mustardé method and frontal muscle flap suspension to correct the ptosis. Postoperatively, the palpebral fissure length was 28 mm on the right and 27 mm on the left, both palpebral fissure height was 8 mm, the inner intercanthal distance was 40 mm, and both of the upper margin reflex distances were 3 mm (Fig. 3a and b).

The results of the first operation were not maintained. The palpebral fissure and inner intercanthal distances regressed. The obtuse deformity of the inner canthus remained. The height of the lateral palpebral fissure remained lower than normal, and both the upper and lower lachrymal points were closed. An ophthalmic examination performed in 2015 revealed the patient’s visual acuity was 20/20, the horizontal fissure length was 21 mm on the right and 22 mm on the left, both palpebral fissure height was 6 mm, the inner intercanthal distance was 48 mm and both of the upper margin reflex distances were 2 mm (Fig. 3c and d).

**Fig. 4:**
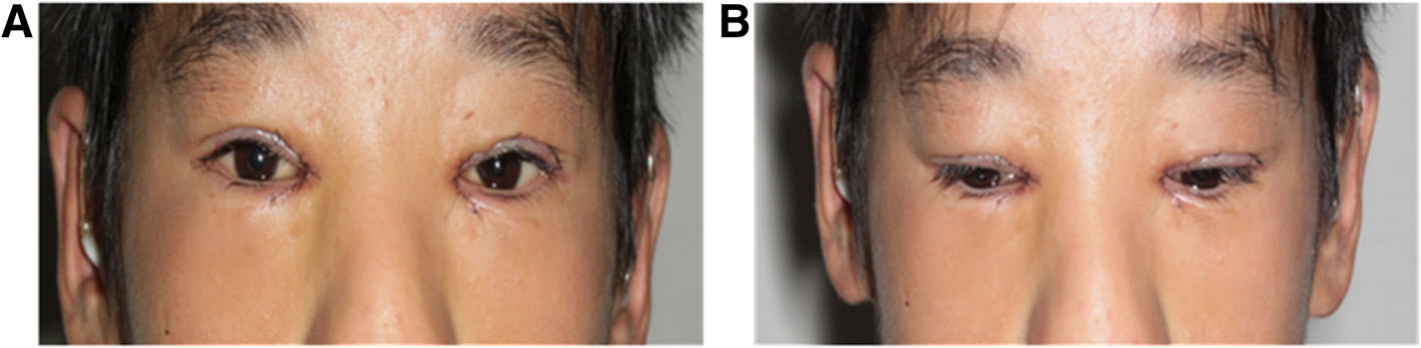
**a**, **b** An examination performed after the second operation in 2015 showed that the horizontal fissure length was 28 mm on the right and 28 mm on the left, both palpebral fissure height was 10 mm, the inner intercanthal distance was 30 mm, and both of the upper margin reflex distances were 4 mm

To further improve the patient’s appearance, the ophthalmologists performed an inner eye canthus Y-V-plasty with frontal flap suspension in 2015 when the patient was 16 years old. The inner canthus deformity was corrected after the second operation. The horizontal fissure length was 28 mm on the right and 28 mm on the left, both palpebral fissure height was 10 mm, the inner intercanthal distance was 30 mm, and both of the upper margin reflex distances were 4 mm (Fig. 4a and b). These results were stable after more than 1 year.

## Discussion

GS is associated with a genetic predisposition and is characterized by epibulbar, aural, facial, and spinal dysplasia in addition to, in some cases, cardiac, renal, and central nervous system anomalies [4]. A diagnosis of GS includes the following clinical manifestations: auricular agenesia, hemifacial microsomia, oblique facial cleft, epibulbar dermoids and/or upper eyelid colobomas, and vertebral anomalies [5, 6]. In addition to these typical malformations, Bowen et al. [7] suggested that affected patients might also have variable abnormalities, such as unilateral or bilateral facial macrosomia, cleft palate, high palatal arch, bifid tongue, bregmatic patency, orbital hypertelorism, small hypophyseal fossa, low hairline, equinus, dumbness, Tetralogy of Fallot, bundle-branch block, lateral ventral syndrome, mild apnoea, epilepsy, hydronephrosis, hydroureterosis, anal atresia, or rectovaginal fistula.

Some patients with GS have clinical findings that overlap with those of other syndromes, such as Treacher Collins syndrome [8], Townes–Brocks syndrome [9], CHARGE syndrome [10], Branchio-oto-renal spectrum disorders [11], and the phenotypic spectrum associated with mutations in EFTUD2 [12, 13]. In the case described here, the patient’s clinical presentations included ocular hypertelorism, hearing loss, and multiple acral joint deformities. Our case was consistent with many previously described cases, including patients 26 and 28 in a study of approximately 51 Oculo-auriculo-vertebral spectrum(OAVS)patients [14]. The clinical manifestations observed in our patient were closely related to GS, of which blepharophimosis with limb deformities is a characteristic manifestation.

We believe that these manifestations are distinctive and recognizable phenotypic entities. However, if a patient’s features are sufficiently suggestive, molecular testing for genes related to these syndromes might be considered in some patients with GS.

In view of his parents’ strong desire for surgical management of his ocular deformities and to have a direct and positive influence aimed at producing psychosocial and social benefits, it was decided that the patient would undergo surgery to improve his facial appearance. The main aim was to correct the inner canthus deformity. The first operation significantly improved the blepharophimosis and shortened the inner intercanthal distance. However, an obtuse deformity of the inner canthus remained. The second operation achieved ideal results. In these types of abnormalities, it is better to correct an inner canthus deformity in the first surgery and to subsequently perform a ptosis surgery. The upper and lower lachrymal point closure accompanied by a lack of tear production that was exhibited by this patient may have been caused by low lachrymal gland secretion, which can represent a compensatory mechanism caused by the deformity. A Schirmer I test revealed a Schirmer of 5 mm, But with 8 s and did not indicate excessive tear production. Because no obvious dry eye syndrome was observed, lachrymal recanalization was not performed. There was no need to treat the patient’s long-term hearing loss or joint deformities after consultation with ear-nose-throat and orthopaedic clinics as neither condition had a significant impact on his quality of life. We recommended that the patient wear hearing aids as usual and that he submit to long-term follow-up observations and symptomatic treatments as needed.

## Conclusion

In this disease, timely detection and intervention can lead to satisfactory surgical outcomes. To obtain a comprehensive assessment, ophthalmology specialists should consult with ear-nose-throat, orthopaedic, neurosurgery, and paediatric clinics, and these entities should plan the best possible interventions that will achieve optimal results for patients with GS. Systemically evaluating patients with GS is important to their diagnosis and enables early intervention to treat the abnormalities that can potentially occur in these patients.

## Abbreviations

GS: Goldenhar syndrome
OAVS: Oculo-auriculo-vertebral spectrum
